# Effects of persistent environmental pollutants on the HPA-axis

**DOI:** 10.1186/1751-0147-54-S1-S17

**Published:** 2012-02-24

**Authors:** Karin Zimmer

**Affiliations:** 1 Section of Experimental Biomedicine, Department of Basic Sciences and Aquatic Medicine, Norwegian School of Veterinary Science, Norway

## Background

Many persistent chemicals which are abundant in nature accumulate through food webs and are transferred to the offspring of pregnant females through the placenta and mother’s milk. Some of these chemicals can adversely affect neurodevelopment, the immune system or act as endocrine disruptors.

Effects of persistent organic pollutants (POPs) on the hypothalamus-pituitary-adrenal (HPA) axis have been relatively poorly studied in comparison with other endocrine axes. However, blood and tissue levels of certain POPs have been shown to correlate with cortisol levels in polar bears and to affect adrenal function in fish and other wildlife [1]. The HPA axis and its end product, cortisol, is not only crucial for adequate responses to stress, it is also involved in normal foetal development, as well as regulation of metabolism, immune function, blood pressure and other bodily functions.

The aim of our research has been to investigate endocrine disrupting effects of different polychlorinated biphenyls (PCBs) and mixtures of POPs. Effects were observed on the adrenal steroidogenesis and foetal development of the adrenal cortex. Special attention was given to the synthesis of cortisol.

## Results

Studies in sheep and goats showed that exposure to PCBs during foetal life and the suckling period caused altered cortisol levels in the blood of both fetuses and adult animals. This indicates that exposure during these sensitive, initial stages of life may have long-term consequences. The findings are important because altered cortisol balance during early life may predispose to development of disease in adulthood, such as diabetes and cardiovascular diseases.

Knowledge about how POPs work and how different POPs act together is important for the assessment of human and animal health risks. Human adrenocortical cells were exposed to different PCB congeners and to POP mixtures. A POP mixture extracted from fish in Lake Mjøsa, Norway, contained high levels of brominated flame retardants; however, this mixture was not more potent in disrupting hormone secretion than a similar POP mixture extracted from fish in another lake with considerably lower levels of these compounds. Thus, brominated flame retardants did not seem to play an important role in altering steroidogenesis in human adrenocortical cells. In the same cells, another POP mixture which was extracted from crude, unprocessed cod liver oil had a pronounced effect on the synthesis of cortisol and sex hormones. However, POPs extracted from commercial cod liver oil, which is frequently consumed as a dietary supplement, was shown to have only limited effects.

## Conclusion

It was concluded that the development of the HPA axis is affected by exposure to PCBs and that cortisol synthesis appears to be a sensitive target for POPs. The effects of POPs on this system were different in *in vitro* and *in vivo* studies and also varied according to life stage *in vivo*. It remains for further studies to assess whether these POP-induced effects are associated with health risks in humans and animals.
